# Literary Notes

**Published:** 1902-02

**Authors:** 


### LITERARY NOTES.

The Maltine Company's Prizes.—Believing that a proper exercise of preventive medicine is of incalculable importance to the human race and desiring to stimulate further research in this line, or at least to disseminate some of the newer ideas so prominently discussed by the medical profession in recent years, we offer two prizes: a first prize of one thousand dollars and a second prize of five hundred dollars, in cash, for the best essays on that subject.

*Conditions of the Competition.*—First.—Essays offered in competition must treat the subject of preventive medicine in its various relations to the welfare of the human race, either treating the topic in its broadest scope as affected by disease, custom, environment, heredity, etc., or from the view-point of the specialist who contends that the most potent factors inimical to mankind result from special conditions which he is enlisted to combat.

Second—In order that there may be no violation of medical ethics and no suspicion of mere commercialism on our part, maltine or any of its combinations must not be mentioned or even indirectly alluded to in the essays.

Third—Competition is open to graduates of all recognised medical colleges.

FOURTH—The essays will be judged by the following gentlemen: Daniel Lewis, A.M., M.D., New York, President New York State Board of Health; Professor of Special Surgery (Cancerous Diseases), Post-graduate Medical School; Surgeon to the Skin and Cancer Hospital; Editor *Medical Review of Reviews.* Charles A. L. Reed, A. M., M. D., Cincinnati, Ex-President American Medical Association; Ex-President American Association of Obstetricians and Gynecologists; Fellow British Gynecological Society. John Edwin Rhodes, A.M., M.D., Chicago, Associate Professor Diseases of the Chest, Throat and Nose, Rush Medical College; Former Professor of Physical Diagnosis and Clinical Medicine, Northwestern University Woman's Medical College, and the prizes awarded in accordance with their decision.

FIFTH—The essays are to consist of at least ten thousand words.

SIXTH—Each competitor is to send us three typewritten copies of his essay by mail in a sealed envelope. These copies are not to be signed by the author, or contain anything which might point to his identity, but are to be signed with a *nom-de-plume.*

SEVENTH—Another sealed envelope shall be sent to us containing this *nom-de-plume* together with the author's name and address. The envelope must be endorsed "For Identification," and will remain sealed until the judges have decided upon the two prize-winning essays, and will then be opened in order that the names of the successful competitors may be ascertained.

EIGHTH—The prize essays and any others which are deemed suitable will be published in a medical journal or journals subject to the approval of the authors.

NINTH—We reserve the right to republish any of these essays in pamphlet form, restricting the circulation to the medical profession.

TENTH—Essays entered in competition must be in our hands by September 1, 1902.

THE MALTINE COMPANY,
8th Avenue and 18th Street, Brooklyn, New York.

---

THE *American Gynecological and Obstetrical Journal* announces its discontinuance in and with its issue for December, 1901. It has struggled against heavy odds from the outset of its career, though it must be confessed it made a strong fight and went down "just outside the breastworks."

It says: The *Journal* shows on its credit sheet a balance due from subscribers and advertisers over and above all indebtedness and expenses. It shows also that during the past ten years over 5,000 subscribers have received and contracted to pay for it who have never paid. It shows, further, that over $30,000 has been lost through unpaid subscriptions by contracts freely entered into by medical men. This exhibits a demoralisation in the medical profession that is shameful, to say the least.

But it also shows that the journalistic field is overcrowded and that men cannot be made to pay for that which they have, perhaps, been overurged to subscribe for.

---

Battle & Company's chart No. 6, illustrating fractures of the long bones, depicts fractures of the patella, showing the separation of the fragments by the rectus and vasti muscles. It is an excellent anatomico-surgical plate.

---

Messrs. P. Blakiston's Son & Co., of Philadelphia, the American agents of the New Sydenham Society, at the request of Jonathan Hutchinson, Esq., F. R. S., secretary of the society, announce the publication of An Atlas of Clinical Medicine, Surgery and Pathology, selected and arranged with the design to afford, in as complete a manner as possible, aids to diagnosis in all departments of practice. It is proposed to complete the work in five years, in fasciculi form, eight to ten plates issued every three months in connection with the regular publications of the society. The New Sydenham Society was established in 1858, with the object of publishing essays, monographs and translations of works which could not be otherwise issued. The list of publications numbers upwards of 170 volumes of the greatest scientific value. An effort is now being made to increase the membership, in order to extend its work.

---

The *Brooklyn Medical Journal*, one of our most respected exchanges, began the 16th year of its publication in a new dress and with a change of form, from standard octavo to double column quarto. It makes a fine appearance in its new outfit and we extend our congratulations. At the same time we always like the standard octavo size the best because it makes the most convenient shape when bound. However, as comparatively few bind a journal, perhaps that is of small consequence.

